# Cyclosporine A Treatment Inhibits *Abcc6*-Dependent Cardiac Necrosis and Calcification following Coxsackievirus B3 Infection in Mice

**DOI:** 10.1371/journal.pone.0138222

**Published:** 2015-09-16

**Authors:** Jennifer Marton, Danica Albert, Sean A. Wiltshire, Robin Park, Arthur Bergen, Salman Qureshi, Danielle Malo, Yan Burelle, Silvia M. Vidal

**Affiliations:** 1 Department of Human Genetics and Complex Traits Group, McGill University, Montreal, Canada; 2 Department of Ophthalmogenetics, The Netherlands Institute for Neuroscience, Amsterdam, The Netherlands; 3 The Center for Host Resistance and the Division of Experimental Medicine, McGill University, Montreal, Canada; 4 Faculty of Pharmacy, University of Montreal, Montreal, Canada; University of British Columbia, CANADA

## Abstract

Coxsackievirus type B3 (CVB3) is a cardiotropic enterovirus. Infection causes cardiomyocyte necrosis and myocardial inflammation. The damaged tissue that results is replaced with fibrotic or calcified tissue, which can lead to permanently altered cardiac function. The extent of pathogenesis among individuals exposed to CVB3 is dictated by a combination of host genetics, viral virulence, and the environment. Here, we aimed to identify genes that modulate cardiopathology following CVB3 infection. 129S1 mice infected with CVB3 developed increased cardiac pathology compared to 129X1 substrain mice despite no difference in viral burden. Linkage analysis identified a major locus on chromosome 7 (LOD: 8.307, P<0.0001) that controlled the severity of cardiac calcification and necrosis following infection. Sub-phenotyping and genetic complementation assays identified *Abcc6* as the underlying gene. Microarray expression profiling identified genotype-dependent regulation of genes associated with mitochondria. Electron microscopy examination showed elevated deposition of hydroxyapatite-like material in the mitochondrial matrices of infected *Abcc6* knockout (*Abcc6-/-*) mice but not in wildtype littermates. Cyclosporine A (CsA) inhibits mitochondrial permeability transition pore opening by inhibiting cyclophilin D (CypD). Treatment of *Abcc6* -/- mice with CsA reduced cardiac necrosis and calcification by more than half. Furthermore, CsA had no effect on the CVB3-induced phenotype of doubly deficient *CypD*-/-*Abcc6*-/- mice. Altogether, our work demonstrates that mutations in *Abcc6* render mice more susceptible to cardiac calcification following CVB3 infection. Moreover, we implicate CypD in the control of cardiac necrosis and calcification in *Abcc6*-deficient mice, whereby CypD inhibition is required for cardioprotection.

## Introduction

Cardiovascular disease (CVD) has emerged as the leading cause of death and morbidity in the world. It is complex and depends on many interacting environmental, genetic, and lifestyle determinants [1]. Although the genetic determinants of CVD remain largely undefined, compelling evidence for the contribution of infectious agents has emerged. Coxsackievirus type B3 (CVB3), a cardiotropic enterovirus, is an important environmental determinant of myocardial necrosis, inflammation, calcification, and fibrosis [2]. Compounded, these can lead to severe outcomes such as myocarditis, dilated cardiomyopathy and death, particularly in young, otherwise healthy individuals [3, 4].

CVB3 infection of cardiomyocytes causes myocardial damage through several mechanisms. The viral lifecycle necessitates membrane lysis to ensure viral propagation. Moreover, virally encoded proteases can also arrest host transcription and translation, cleave cardiac structural proteins, and promote apoptosis and necrosis [5, 6]. Overexpression of either 2A^pro^ or 3C^pro^, for example, results in both caspase 3 activation and in the release of cytochrome c from mitochondria, leading to dramatically reduced cell viability [7, 8]. Observed increases in the production of (ROS) following infection may also lead to increased permeability of the mitochondrial outer membrane and permeability transition pore (mPTP) thereby encouraging both apoptosis and necrosis [9, 10]. Lastly, endoplasmic reticulum (ER) stress can also lead to large calcium fluxes from the ER to the mitochondria, further sensitizing the mPTP [11, 12]. Indirectly, infection leads to the recruitment of immune cells whose presence can be detrimental; immune cell killing of infected cells and the release of cytokines that are negatively inotropic contribute to myocardial damage [2, 3]. Cardiac injury is currently irreversible and cardiomyocytes that die as a result of CVB3 infection are replaced with fibrotic or calcified tissue [3, 13]. This drastically compromises heart contractility.

The development of prophylactic therapies against CVB3 has been unsuccessful and treatments are currently symptom-based [14]. The high mutation rate of the viral polymerase coupled to the large number of serotypes have hindered traditional vaccine and antiviral approaches [15]. Treatments that reduce cardiac damage independently of virus may prove useful but none have been consistently successful. The literature is divided, for example, on the benefit of immunosuppression in reducing the cardiac inflammation and necrosis associated with myocarditis [16–18]. This underscores the idea that there are other interacting factors that dictate whether a given treatment will be efficacious.

The importance of host genetics in modulating the severity of CVB3-dependent cardiopathology is made clear in the mouse model where environmental, lifestyle, and viral determinants can easily be kept constant. Inbred and genetically engineered mice infected with CVB3 develop a spectrum of pathologies ranging from asymptomatic to acute myocarditis to dilated cardiomyopathy [19, 20]. We therefore hypothesized that, by identifying host genes that modulate cardiopathology without affecting viral fitness, we could more effectively target treatments and ultimately prevent or diminish irreparable heart damage. Here, we utilized a forward genetic approach in the mouse to show that *Abcc6*, an ABC transporter of ATP expressed primarily in the liver and kidney [21, 22], modulates CVB3-dependent cardiopathology despite no effect on viral burden. We further demonstrate that treatment with cyclosporine A (CsA) diminishes CVB3-dependent cardiac calcification and necrosis selectively in *Abcc6* knockout mice. This occurs via its inhibition of cyclophilin D (CypD), a regulator of mitochondrial permeability transition pore opening.

## Materials and Methods

### Mice

Inbred 129S1/SvImJ (129S1), 129X1/SvJ (129X1) and C57BL/6 (B6) mice were purchased from Jackson Laboratories (002448, 000691 and 000664 respectively) and maintained as breeding colonies at McGill University. (X1 x S1)F2 progeny were obtained by brother-sister mating of (X1 female x S1 male)F1 mice. *Abcc6* ^-/-^ (*Abcc6* KO) mice were a generous gift from A. Bergen and had been backcrossed to C57BL/6 mice for 14 generations prior to acquisition[23]. *Abcc6* KO mice were crossed to C57BL6 wild type (WT) mice to generate *Abcc6* ^+/-^ (*Abcc6* Het) mice. *Abcc6* Het mice were intercrossed to generate KO, Het, and WT mice for phenotyping. (S1 x *Abcc6* KO)F1 and (S1 x *Abcc6* WT)F1 were derived by intercrossing 129S1 with *Abcc6* KO and WT respectively. CypD deficient B6;129-*Ppif* ^tm/Jmol^/J (*CypD* KO) were purchased from the Jackson laboratory (#009071). *CypD* KO mice were intercrossed with *Abcc6* KO mice to generate *Abcc6* ^+/-^ / *CypD* ^+/-^ mice. These were crossed to either *Abcc6* KO or WT mice to generate *Abcc6* ^-/-^ / *CypD* ^+/-^ and *Abcc6* ^+/+^ / *CypD* ^+/-^ mice respectively. Both of these were intercrossed to generate *Abcc6* ^-/-^ / *CypD* ^-/-^ (DKO) and *Abcc6* ^-/-^ / *CypD* ^+/+^ (SKO) littermates and *Abcc6* ^++^ / *CypD* ^-/-^ and *Abcc6* ^+/+^ and *CypD* ^+/+^ littermates. All animals were maintained at the Goodman Cancer Centre animal facility at McGill University. The McGill University Animal Care Committee (UACC) approved all animal housing and experimental procedures. The UACC holds a certificate of “Good Animal Practice” from the Canadian Council on Animal Care.

### Virus

The CVB3 H3 plasmid [PMID: 8892902] was kindly provided by the laboratory of Dr Kirk Knowlton (University of California, San Diego). The plasmid was expressed by transfection in COS-7 cells and the resulting virus was propagated once in HeLa cells. The HeLa cells and culture medium were harvested, freeze-thawed three times, and centrifuged at 1000xg to remove cellular debris. The supernatant was aliquoted and frozen at -80°C. A new aliquot was thawed for each infection to ensure experimental consistency.

### Infection, Treatment, Organ Harvest

Mice 7 to 8 weeks of age were inoculated intraperitoneally with CVB3 diluted in sterile PBS. Mice on a 129 background were inoculated with a dose of 500 plaque forming units (pfu)/g whereas mice on the comparatively susceptible B6 background [24] were inoculated with a dose of 50 pfu/g and monitored daily for weight loss and signs of distress. We aimed to phenotype at least 5–10 mice per group. This sample size was chosen based on empirical evidence that it provides enough statistical power to detect biologically relevant differences in phenotype. Mice were sex matched where possible to block the potential effect of sex as a confounder in downstream statistical analyses. Mice that lost 15–20% of their initial body weight, had a hunched posture, and reduced mobility prior to experimental endpoints were euthanized humanely with CO_2_. In our model, mice that do not lose weight by day 4 post infection have very little or undetectable virus, regardless of strain, suggesting that this is due to a faulty infection rather than to a resistance mechanism. To avoid biasing results due to failed or incomplete infections, we omitted mice that did not lose at least 10% of their initial body weight by day 4 post-infection. For each experiment, S1 Table outlines the number of mice that were infected, mice that died, and mice that were omitted because they did not lose weight. These are summarized by genotype, sex, and treatment when applicable. Mice used to quantify cardiomyocyte necrosis were injected with 10mg/kg body weight of Evan’s blue dye (EBD) 24hrs prior to the experimental endpoint. Treated mice were dosed daily with cyclosporine-A (CsA: 10mg/kg/day), FK506 (1 and 10 mg/kg/day) or vehicle (1% DMSO in corn oil) by oral gavage. At each experimental endpoint, mice were sacrificed with CO_2_ and hearts were perfused with 10mL of PBS before organ excision unless otherwise indicated. Mice of differing genotypes were cohoused to avoid cage effects and cages were randomly assigned to treatment groups. Collected samples were processed blindly.

### Phenotyping

Myocarditis Score: Excised hearts were fixed in 10% formalin and sent to the histology lab in the Department of Pathology at the University of Ottawa for embedding, sectioning, and staining. Samples were paraffin embedded and 5μm sections were stained with H&E or alizarin. Microscopic determination of myocarditis score was determined with H&E stained sections using previously established criteria. Briefly, each section was assigned a myocarditis score ranging from 0 (least severe) to 4 (most severe). ‘0’ was assigned to sections that had no inflammation. ‘1’ was assigned to sections with very mild and focal inflammation. ‘2’ was assigned to sections with multiple small to medium sized focal inflammation. ‘3’ was assigned to sections with large focal inflammation. ‘4’ was assigned to sections having coaslescent inflammation that is spread throughout the tissue (representative images in [25]). Microscopic determination of calcification was determined with alizarin stained sections. Red staining was indicative of cardiac calcification.

#### Calcium Quantification

Excised hearts were decalcified by incubating at room temperature in 0.6N HCl for 24 hours. The concentration of calcium in the decalcifying medium was measured using BioVision’s calcium colorimetric assay according to instructions provided by the manufacturer. Readings were obtained using a SpectraMax 190 microplate reader set at 575nm. All readings were taken within the window of fluorochrome stability (30 minutes) and normalized to heart weight. Calcification is reported as the mean calcium concentration +/- SD.

#### Viral Titer

Tissue samples were homogenized in 1mL unsupplemented DMEM. Serial tenfold dilutions of homogenate were applied to confluent HeLa (ATCC: CCL-2) cell monolayers in triplicate in 12 well plates. Plates were incubated at 37°C and 5% CO_2_ for one hour to allow viral entry. Each well was then overlain with a 1:1 ratio of DMEM supplemented with 20% FBS, 2% penicillin/ streptomycin and 0.5% agarose. Assays were incubated at 37°C and 5% CO_2_ for three days after which they were fixed with 10% phosphate buffered formalin and stained with 0.5% crystal violet in 70% ethanol. Plaques were counted and titers were normalized to heart weight. Viral titer is reported as mean +/- SD.

#### Quantification of Myocyte Necrosis

Excised hearts were embedded in optimal cutting temperature compound and snap frozen in a dry ice-chilled isopentane bath. Embedded samples were cryo-sectioned (5μm) at the GCRC histology facility at McGill University. Low magnification (10X) images were generated using a 450/480nm fluorescence filter on a Zeiss Axioscope II microscope. Heart sections were photographed in their entirety and assembled using DoubleTake. Fluorescent areas were indicative of EBD intercalation and were quantified as a percentage of total area using ImageJ. Briefly, images were converted to binary form (black and white pixels). The ratio of white pixels to total pixels was used to define percent area. Myocyte necrosis is reported as mean +/- SD.

#### RNA Extraction and Microarray

Total RNA was isolated from excised hearts and livers using the TRIzol reagent (Ambion) according to the protocol outlined by the manufacturer. RNA for microarray analysis was further purified using Qiagen’s RNeasy Kit (Qiagen) according to the protocol outlined by the manufacturer. RNA quality assessment, cDNA synthesis, and hybridization were performed at McGill University’s Genome Quebec Innovation Centre. RNA quality was assessed using Agilent’s Bioanalyzer 2100. All RNA preparations had RNA Integrity Number (RIN) scores greater than 8. cDNA samples were randomly hybridized to Illumina MouseWG-6 version 2.0 Expression BeadChipsTM. RNA was extracted from three infected 129S1 and 129X1 hearts and three uninfected 129S1 and 129X1 hearts and livers. Raw data are available online through NCBI GEO under accession number GSE44706. Normalization was performed using the log2 and RMA configuration of Lumi in FlexArray version 1.6.1. Principal component analysis figures (S3 Fig) were generated using the prcomp function in that “stats” package of R. Genes for consideration in functional clustering and pathway enrichment were filtered as previously described [26] with a few alterations to account for the genetic similarity between 129S1 and 129X1. Briefly, genes considered differentially expressed between strains upon infection were identified according to the following criteria: (1) expression values greater than the 40^th^ expression percentile in at least one genotype/condition; (2) expression fold change upon infection of at least 1.5 fold (cyberT < 0.05) in at least one strain; (3) two-way ANOVA (strain * infection) less than 0.05; and (4) absolute difference of differences between strain means (|((μ_129S1_ − μ_129S1 infected_) − (μ_129X1_ − μ_129X1 infected_))|) greater than 0.3 (corresponding to the top 4% of absolute difference of differences). Pathway enrichment was performed using the Database for Annotation, Visualization and Integrated Discovery (DAVID) v6.7 (http://david.abcc.ncifcrf.gov/). The gene lists defined above were uploaded and functional clusters having Benjamini *p* values<0.05 were considered significantly enriched.

#### Mitochondrial Isolation and Swelling Assay

Mitochondria and swelling assays were performed as previously described[27]. Swelling assay results are reported as the change in mean optical density +/- SD.

#### Transmission electron microscopy

Mice were anesthetized with a 100 μL intraperitoneal injection of pentobarbital (54.7mg/mL). Anesthetized mice were gravity perfused with lactated Ringer’s followed by fixative (2.5% glutaraldehyde in 0.1M cacodylate buffer). Excised hearts were processed, stained, and sectioned at McGill University’s Facility for Electron Microscopy Research. Necrotic foci were identified visually by bright field examination of toluidine-stained thick sections (0.5mm) based on the pale staining and increased granularity of the cytoplasm (S5 Fig). Corresponding thin sections (0.1 mm) were imaged on an FEI Tecnai 12 120kV electron microscope with an AMT XR80C CCD camera system. Necrotic foci were further classified into three stages (early, mid, late) according to the presence and intactness of mitochondria and to the plasma membrane integrity (S5 Fig). *Abcc6* KO and WT mice (n = 4 / group) were either left uninfected or infected for 6 days with 50pfu/g CVB3. Necrotic foci from two thin sections per infected mouse were analyzed. Mitochondria within necrotic foci were characterized according to several physical characteristics: disruption of cristae, fusion/fission abnormalities, hydroxyapatite deposition, and abnormal shape (See results section)

### Genotyping, Sequencing and Haplotype Analysis

Genomic DNA was prepared from the tails of individual mice. (129X1 x 129S1)F2 DNA was used for custom array genotyping on the Sequenom® iPlex® Gold platform. This was performed at McGill University’s Genome Quebec Innovation Centre using 164 polymorphic SNPs spaced at a frequency of 1 SNP/10Mbp where possible (S2 Table). Additional microsatellite markers used to increase mapping resolution were selected from the Mouse Genome Informatics Database (S2 Table). Genotypes were determined using a standard PCR protocol followed by separation on 3% agarose gels. *Abcc6* and *CypD* genotypes were determined by PCR and gel electrophoresis using primers as previously described[23, 28]. The 14^th^ exon of *Abcc6* was Sanger sequenced at McGill University’s Genome Quebec Innovation Centre using gDNA from 129S1 and 129X1 animals. The following primers were used: ACCCCCAGTGAACAGAGTTG, CCCCTACACTGATGTGCTGA. 129S1, 129X1, and C3H/HeJ haplotypes were determined using the online resource Mouse Phylogeny Viewer (https://msub.csbio.unc.edu/).

### Statistics

Built-in statistics packages in Prism and DAVID were used to evaluate data significance. Statistical tests were chosen to reflect experimental design as indicated in the figure legends. Corrected P values less than 0.05 were deemed significant. Linkage analyses were performed using R/QTL. Non-parametric and binary QTL scans were used to calculate the logarithm of odds (LOD) score for inflammation and calcification respectively. Sex was included as an additive and interactive covariate to determine its effect on each QTL. Empirical significance for each model was established using 10,000 phenotype permutations.

## Results

### A single, highly penetrant locus on chromosome 7 controls CVB3-induced cardiac calcification and inflammation

Inbred strains of mice are a valuable tool for evaluating the impact of naturally occurring genetic variation on a given phenotype. The 129 substrains of mice are quite similar genetically (S1 Fig) [29] but nonetheless differ phenotypically[30]. We aimed to exploit this decreased genetic variation in order to increase our likelihood of identifying causative gene(s) and genetic variant(s) that could impact on the host response to CVB3 infection.

**Fig 1:**
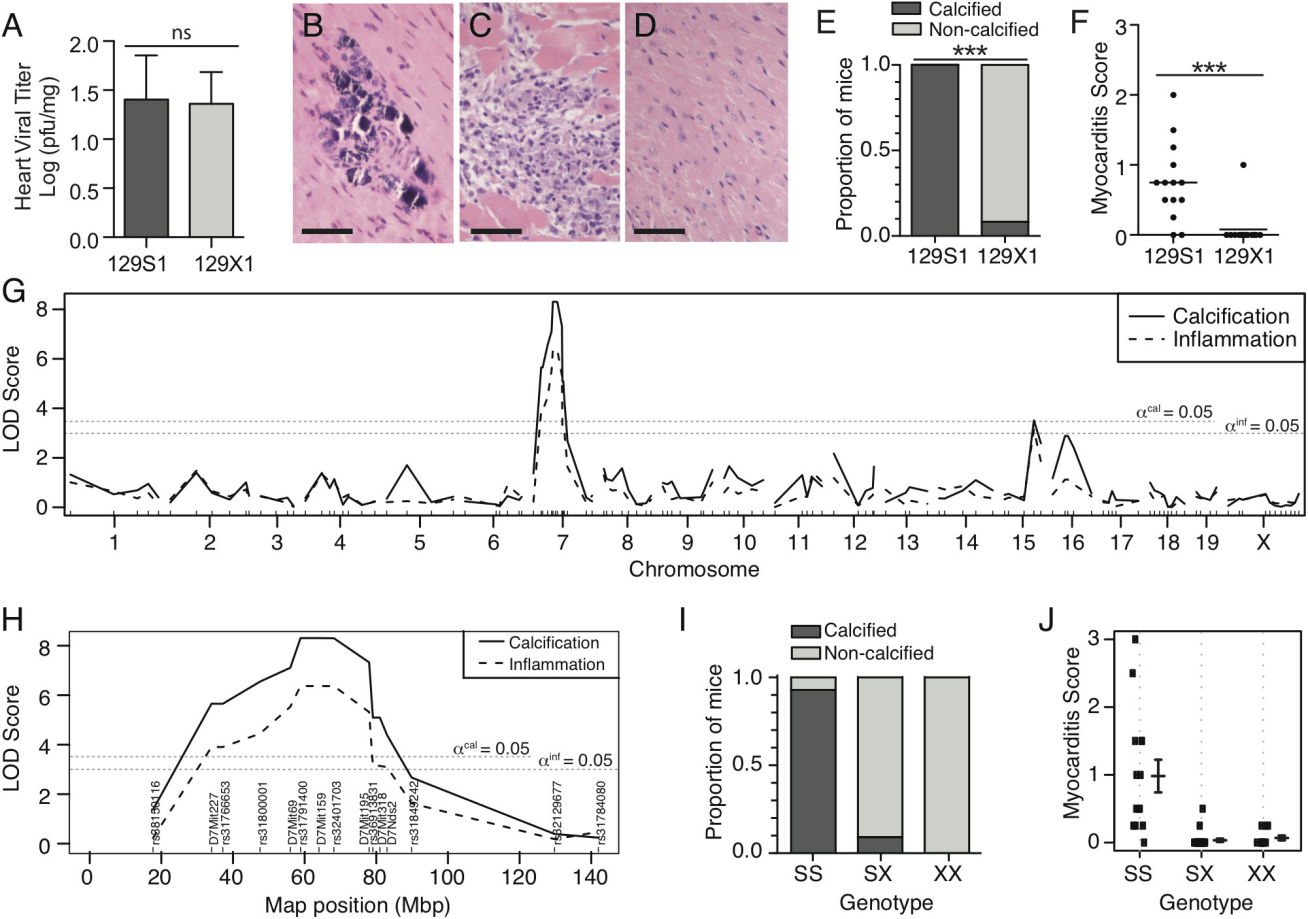
Differential susceptibility to viral myocarditis is controlled by a chr. 7 locus. Cardiac viral titer and histopathology were examined in 129S1 and 129X1 mice infected with 500pfu/g CVB3. Viral titer was equal between both strains (A). 129S1 mice developed calcified lesions (B, E) and increased inflammation (C, F) in response to CVB3 infection. 129X1 mice very rarely presented with either (D, E, F). Analysis of phenotypic segregation and linkage mapping in an (129S1 x 129X1) F2 population identified a common, highly significant locus for each phenotype (Calcification: LOD = 8.307, P<0.0001; Inflammation: LOD = 6.370, P<0.0001) (G). Peak linkage for both traits occurred on Chr. 7 between markers D7Mit69 (56.3 Mbp) and rs32401703 (68.2 Mbp) (H). Recessive inheritance of homozygous S1 alleles conferred susceptibility to both phenotypes (I,J). Statistical tests were chi-squared test and student’s t test for panels E and F respectively. *** *P*<0.001. Bar = 50m.

We first evaluated heart viral titer in 129S1 and 129X1 animals. No significant differences were observed (Fig 1A). Despite this, 129S1 mice developed calcified lesions within inflammatory foci (Fig 1B,1C, 1E and 1F). This contrasted with the lack of pathology observed in 129X1 mice (Fig 1D, 1E and 1F). The effect of sex was minimal compared to the effect of strain (Two-way ANOVA: Calcification: strain = 83.42%, sex = 0.69%; Inflammation: strain = 48.1%, sex = 0.05%). We pursued linkage analysis in a cohort of (129X1 x 129S1)F2 progeny in order to identify genes and genetic loci that control cardiopathology independently of viral titer. The F2 cohort was infected with CVB3, screened for histological presence of calcification and inflammation, and genotyped at 164 polymorphic loci (S2 Table). A single, highly-significant quantitative trait locus (QTL) was observed for each phenotype (Calcification: LOD = 8.307, P<0.0001; Inflammation: LOD = 6.370, P<0.0001) and both localized to an overlapping region of chromosome 7 (peak: 58.8Mbp – 64.0 Mbp). (Fig 1G and 1H). Inheritance of homozygous 129S1 alleles conferred susceptibility to both phenotypes (Fig 1I and 1J). Including sex as either an additive or interacting covariate had no significant effect on either QTL (calcification: p^additive^ = 0.417, p^interactive^ = 0.513, inflammation: p^additive^ = 0.488, p^interactive^ = 0.560).

**Fig 2:**
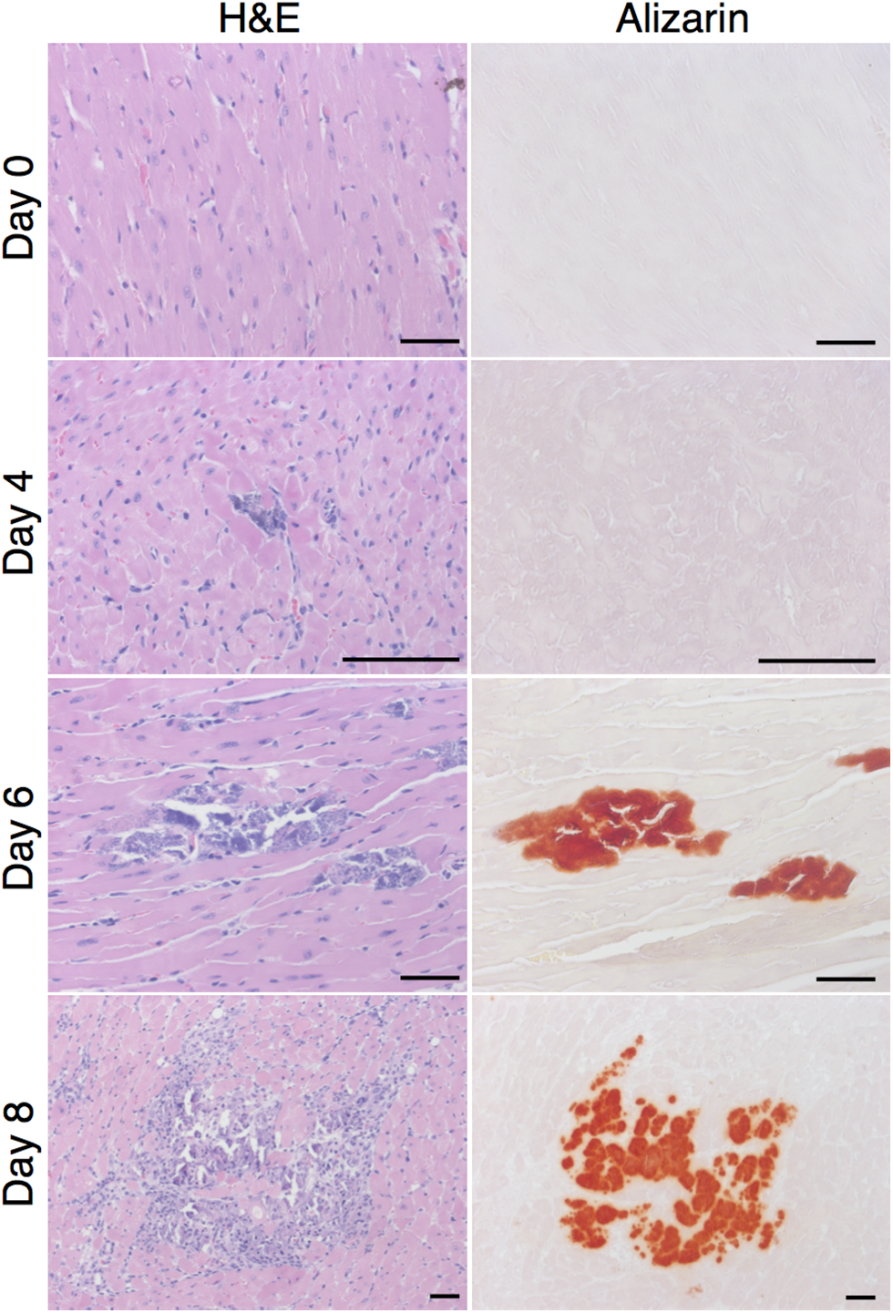
Spatio-temporal relationship between calcification and inflammation following CVB3 infection. Presence and location of calcification and inflammation were assessed at days 0, 4, 6, and 8 post infection in 129S1 mice using alizarin and H&E staining respectively. Normal histology was observed at day 0. Non-calcified lesions were observed 4 days post infection. These expanded in size and number by day 6 post infection at which time calcification became apparent. By day 8 post infection, inflammation was observed and colocalized completely with calcified lesions. Bar = 100μm.

To evaluate the relationship between cardiac calcification and inflammation, we assessed the presence and severity of the two phenotypes in 129S1 mice at several time points post infection. These were evaluated histologically at days 0, 4, 6, and 8 post infection using the calcium-specific alizarin red stain and H&E respectively. Uninfected mice showed no signs of either phenotype (Fig 2). At day 4 post infection, single cell lesions became visible. These expanded by day 6 post infection and stained positively for calcium. By day 8 post infection, inflammatory cells were observed within the calcified lesions. Calcification was observed earlier than inflammation and, when both phenotypes were present, they colocalized completely (Fig 2).

### *Abcc6* controls CVB3-induced cardiac calcification

**Fig 3:**
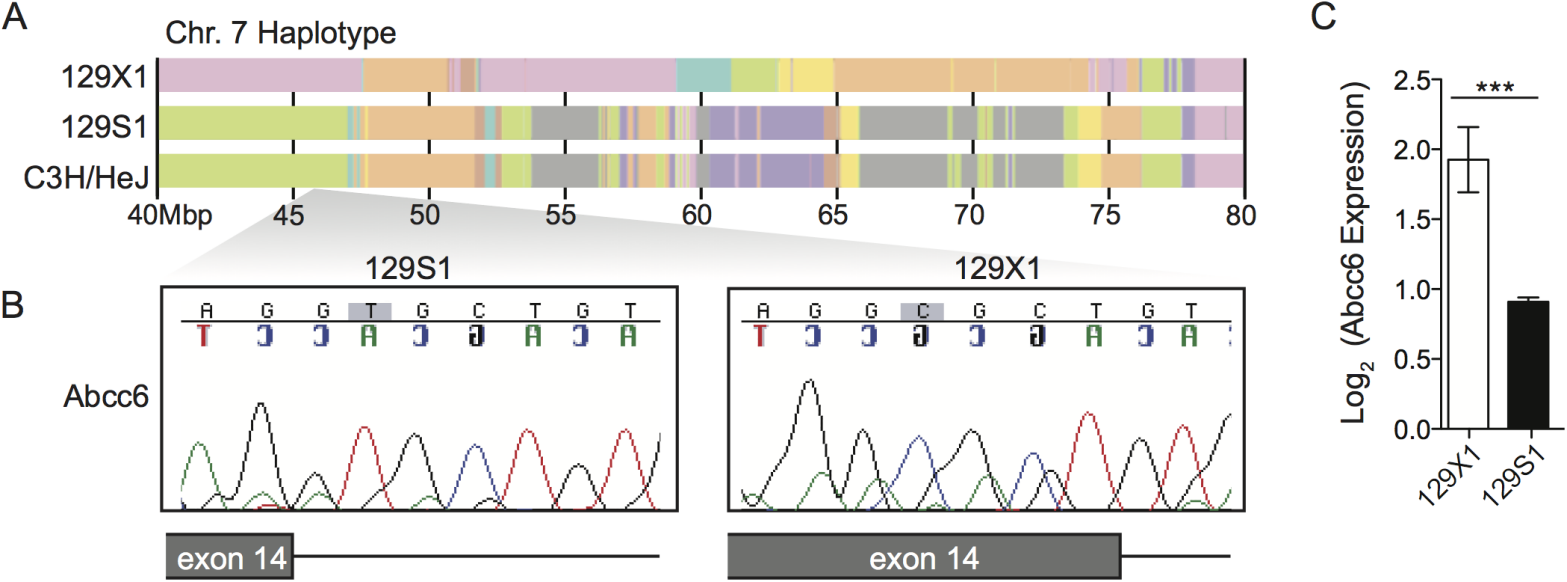
*Abcc6* is a promising candidate gene. (A) 129S1 mice share a common haplotype with C3H/HeJ in the chr.7 QTL interval. (B) Both C3H/HeJ [32] and 129S1 mice possess a mutation in exon 14 of *Abcc6* that creates a new splice donor site and causes a 5 bp deletion. This mutation is absent in 129X1 mice and causes a twofold decrease in *Abcc6* expression in 129S1 livers (C). The statistical test used was a student’s t test. ***P<0.001.

**Fig 4:**
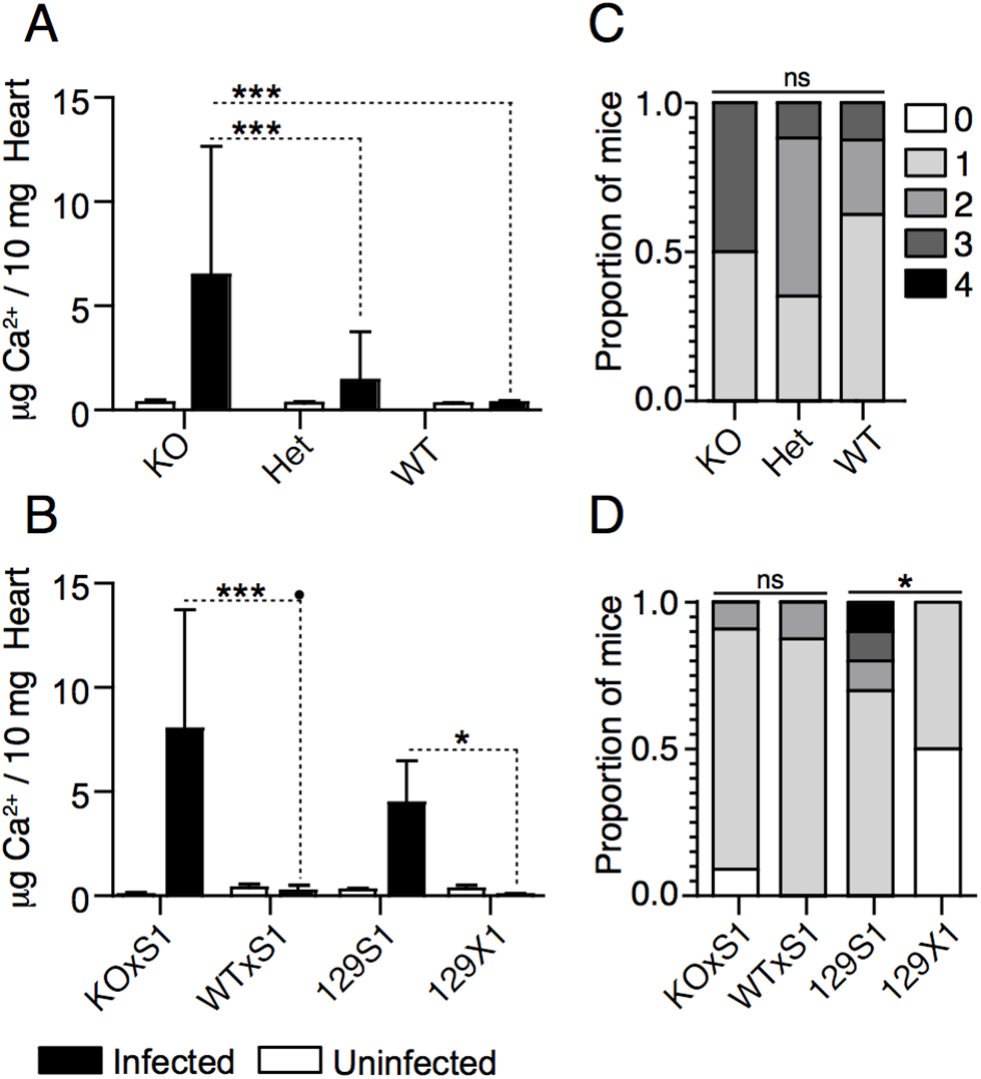
Causative gene identification by allelic complementation tests. Cardiac calcification was quantified in *Abcc6* KO, Het, WT, (*Abcc6* KO x 129S1)F1, (*Abcc6* WTxS1)F1, 129S1, and 129X1 mice before infection and at day 8 post-infection. *Abcc6* KO, Het, and WT mice were infected with 50pfu/g CVB3; the rest were infected with 500pfu/g CVB3. The concentration of calcium in uninfected mice was equal between all strains (A, B). At day 8 post-infection, increased calcification was consistently observed only in *Abcc6* KO, (*Abcc6* KO x 129S1)F1, and 129S1 mice (A,B). The severity of inflammation was evaluated in the same mice. Although differences between 129S1 and 129X1 were apparent, inflammation severity did not differ significantly between the other groups (C, D). The statistical test for panels A and B was a two-way ANOVA followed by Bonferonni post tests. The statistical test for panels C and D was a one-way ANOVA followed by a Dunn’s post-test. *: P<0.05, ***: P<0.001, ns: not significant.

*Abcc6* was a promising candidate gene because of its genomic location (chr 7: 45.9Mbp) and its known role in dystrophic cardiac calcification. Meng et al showed that a splice site mutation in *Abcc6* (c.1866C>T, p.R621C, rs32756904) causes dystrophic cardiac calcification in C3H/HeJ mice*[31]*. The mutation creates a new splice donor site, which causes a 5bp deletion, leads to a premature stop codon and results in a 60% reduction in protein expression [32]. Interestingly, genetic contamination of 129S1 DNA with C3H/HeJ DNA has been described [33]. Moreover, C3H/HeJ and 129S1 mice share a common haplotype, which is distinct from the 129X1 haplotype, in the chr. 7 QTL region (Fig 3A). We therefore sequenced *Abcc6* in 129S1 and 129X1 animals and found that 129S1 mice possess the mutant allele whereas 129X1 mice possess the wildtype allele (Fig 3B). To determine whether this mutation affects *Abcc6* expression, we measured mRNA levels in 129S1 and 129X1 livers. *Abcc6* mRNA expression was reduced by twofold (P = 5.15e-08) in 129S1 mice compared to 129X1 mice (Fig 3C). This was comparable to the reduction in protein expression observed in the C3H/HeJ mice. Given this, we assessed whether *Abcc6* was the gene underlying our locus using *Abcc6* KO mice (Fig 4A and 4C) and genetic complementation (Fig 4B and 4D). We first evaluated calcification in *Abcc6* KO, Het and WT mice at days 0 and 8 post infection. No genotype-dependent differences in calcification were observed in uninfected mice (Fig 4A). Upon infection, levels of calcium in *Abcc6* WT hearts remained constant with infection whereas levels in *Abcc6* KO hearts increased drastically with infection (Fig 4A). We next evaluated calcium levels in two genetic complementation groups, (*Abcc6* KO x 129S1)F1 and (*Abcc6* WT x 129S1)F1 mice, at days 0 and 8 post infection (Fig 4B). Increased calcification was observed in (*Abcc6* KO x 129S1)F1 but not in (*Abcc6* WT x 129S1)F1 mice (p<0.001), thereby confirming that *Abcc6* is responsible for the CVB3-induced calcification in 129S1 mice. Interestingly, inflammation severity remained unaffected by genotype in both experiments suggesting a role for genetic background in modulating this phenotype (Fig 4C and 4D).

### *Abcc6* impacts the extent of cardiomyocyte necrosis following CVB3 infection despite no effect on viral replication

**Fig 5:**
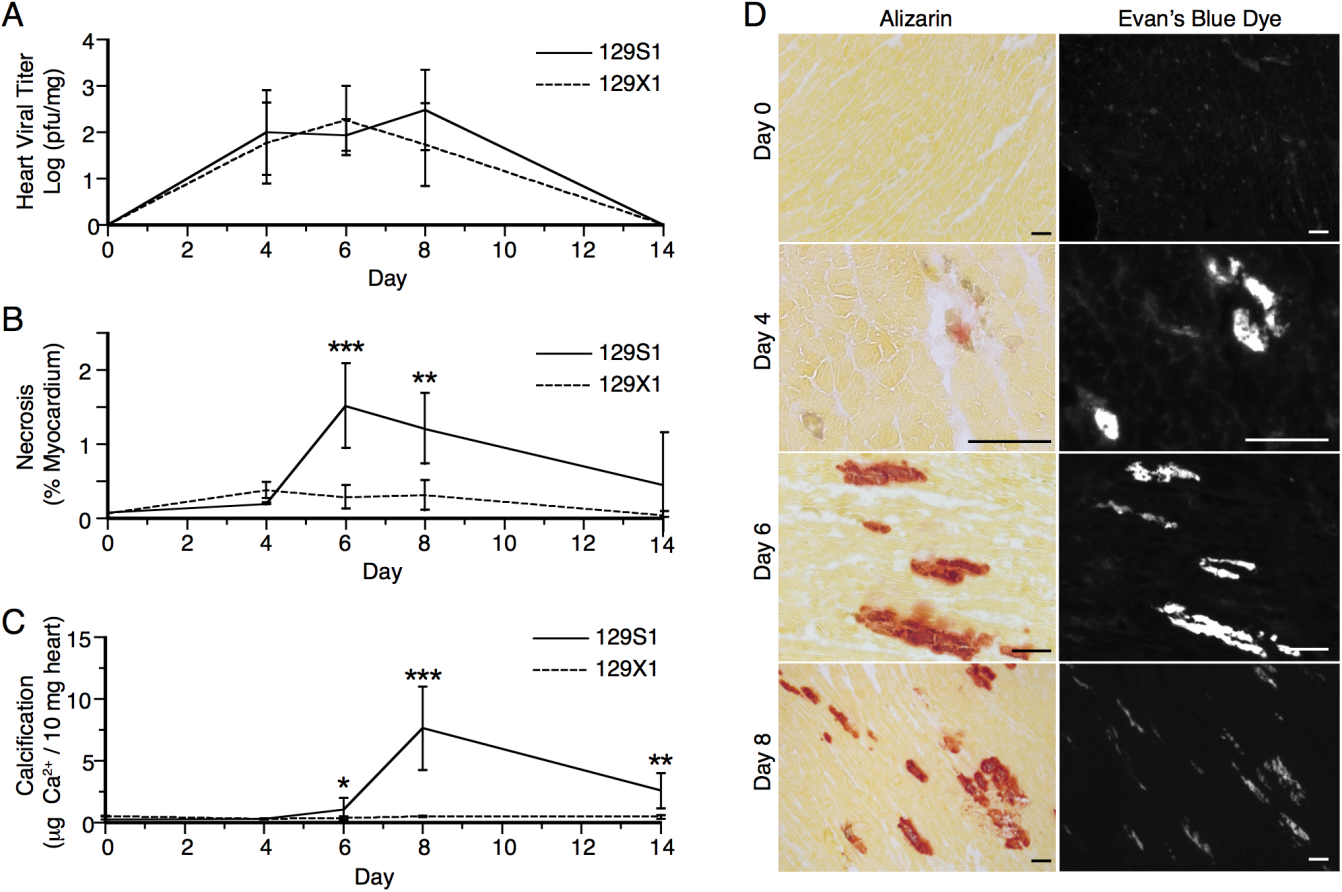
Impact of Abcc6-deficiency on the kinetics of viral titer, necrosis, and calcification. The temporal relationship between viral replication (A), necrosis (B), and calcification (C) was evaluated at days 0, 4, 6, 8, and 14 post-infection with 500pfu/g CVB3 in 129S1 and 129X1 mice. Viral replication did not differ significantly between strains at any time point post-infection (A). Quantitatively significant differences in necrosis and calcification were observed by day 6 post-infection (B,C). The spatial relationship between necrosis and calcification was assessed at days 0, 4, 6, and 8 post-infection using Evan’s blue incorporation and alizarin staining respectively (D). Non-calcified necrotic lesions were apparent by day 4 post-infection. Complete co-localization of necrotic and calcified lesions was observed at days 6 and 8 post-infection. The statistical test for panels A-C was a two-way ANOVA followed by Bonferonni post tests. *: *P*<0.05, **: *P*<0.01, ***: *P*<0.001. Bar = 100mm.

No differences in heart viral titer were observed between 129S1 and 129X1 mice at day 8 post-infection. However, differences in cardiac calcification were observed. As dystrophic cardiac calcification normally occurs following cell death, we examined the kinetics of viral replication, necrosis, and calcification. These were assessed at days 0, 4, 6, 8, and 14 post infection in 129S1 and 129X1 mice. As expected, viral titer did not differ significantly between 129S1 and 129X1 mice at any time point post infection (2-way ANOVA >0.05) (Fig 5A). Interestingly, though, cellular necrosis was significantly higher in 129S1 mice at days 6 and 8 post infection (2-way ANOVA <0.001) (Fig 5B). Calcification followed both viral replication and cellular necrosis and colocalized completely with necrotic areas (Fig 5C and 5D). To exclude the possibility that these observations arose due to an independent background effect, we evaluated viral titer and necrosis in *Abcc6* KO and WT/Het mice at day 8 post-infection. Increased necrosis despite equal viral replication remained evident only in the *Abcc6* KO mice (S2 Fig).

### Global transcriptome analyses implicate mitochondria in *Abcc6*-dependent, CVB3-induced necrosis/calcification

**Fig 6:**
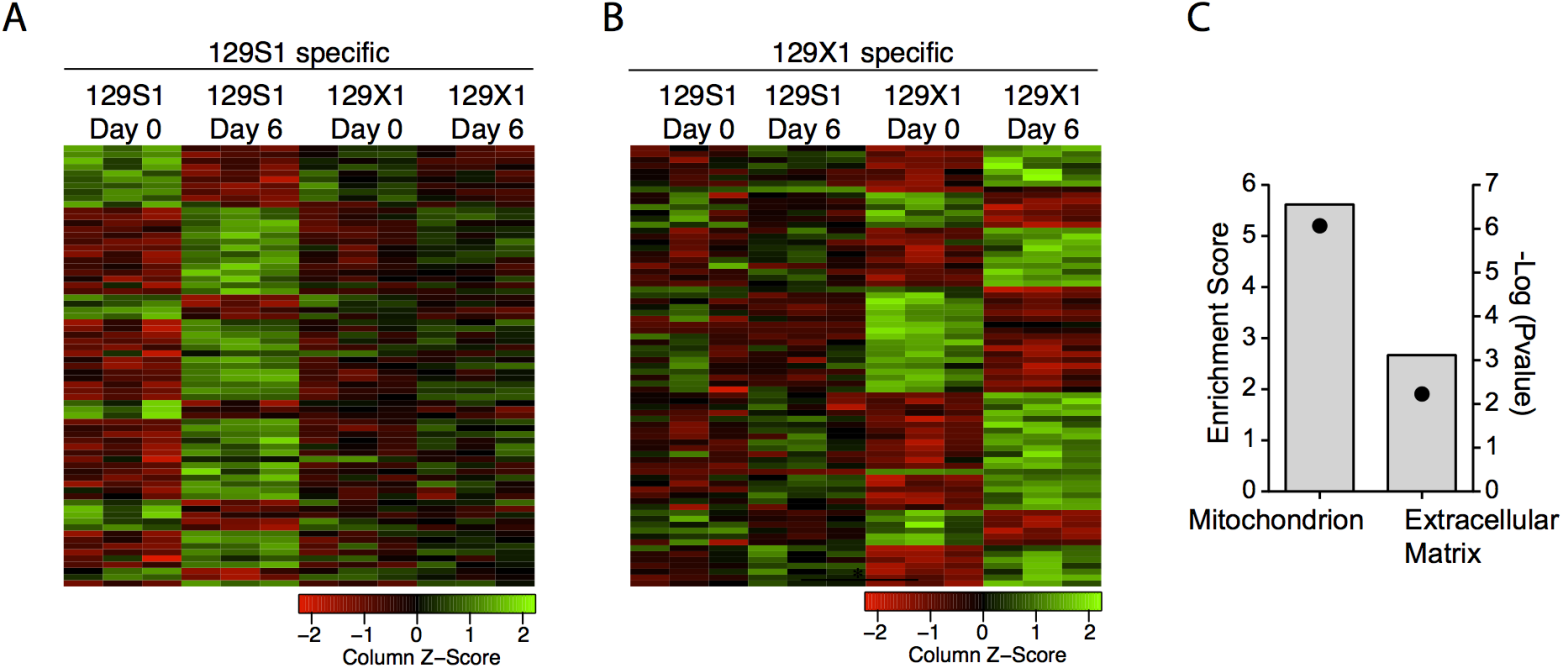
Identification of transcriptional signatures of calcification using microarrays. Gene expression data was assessed before infection and at day 6 post-infection with 500pfu/g CVB3 in 129S1 and 129X1 mice using microarrays. Genotype-specific expression changes were determined. The heatmaps represent 129S1 and 129X1-specific transcriptomic signatures of infection (A, B). Functional clustering of these genes using DAVID identified significant enrichment of two clusters: mitochondrion and extracellular matrix (C). Bars represent the enrichment score and points represent the–log (Bonferonni pvalue).

The increased necrosis despite equal viral titer observed in *Abcc6* deficient mice suggested the existence of a pathologic host response to infection. We used global transcriptomic profiling to provide a snapshot of ongoing host responses in order to identify calcification-specific pathway perturbations. Microarray analyses were conducted on heart tissue from 129S1 and 129X1 mice at days 0 and 6 post infection. The time points were chosen in an attempt to capture ongoing calcification processes occurring independently of inflammation (Fig 2). The first principal component (PC1) explained the majority (59.1%) of expression variance and was associated with infection status, which explained 97.4% (p<0.0001) of the variation in PC1. PC1 was mainly characterized by an induction of antiviral gene expression programs in both strains of mice (S3 Fig). The second principal component (PC2) explained 10.8% of overall expression variance and was significantly associated with strain, which explained 44.0% (p = 0.02) of the variation in PC2 (S3 Fig). To identify and characterize strain-specific expression changes, genes that were up or down regulated with infection differently between 129S1 and 129X1 mice were identified (Fig 6A and 6B). Functional clustering of these genes using DAVID identified significant enrichment of two clusters: mitochondria (p = 9.0x10-7) and extracellular matrix related genes (p = 6.1x10-3) (Fig 6C). Changes in the expression of genes associated with the extracellular matrix likely reflect differences in wound healing responses following necrosis. As such, they likely represent downstream repercussions of calcification rather than causative processes. Given this and the well-established link between mitochondria, calcium overload, and necrosis^23^, we pursued the role of mitochondria in *Abcc6*-dependent cardiopathology.

### *Abcc6*-dependent differences in mitochondrial response following infection

Given the increased necrosis observed in Abcc6 deficient mice and the role of the mPTP in regulating necrosis[28], we hypothesized that differential regulation of mPTP opening might be implicated in *Abcc6*-dependent cardiopathology. We performed *ex vivo* swelling assays on mitochondria isolated from uninfected and infected *Abcc6* KO and WT mice. No genotype-dependent differences were observed in either the absence of calcium or with the addition of 400uM calcium (S4 Fig).

**Fig 7:**
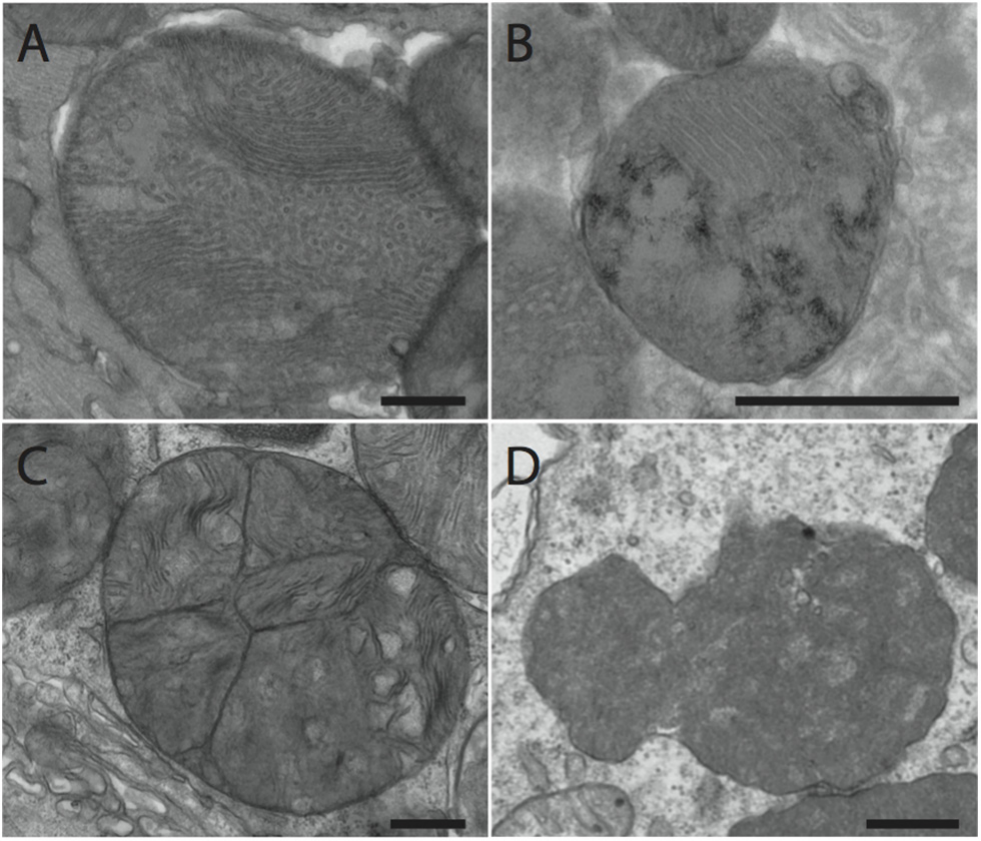
Representative images of abnormal mitochondria used to categorized TEM images from *Abcc6* KO and WT CVB3-infected hearts. Representative images were taken from heart sections from *Abcc6* KO mice infected with 50pfu/g CVB3. (A) Disrupted cristae. (B) Fusion/fission events. (C) Hydroxyapatite deposition. (D) Abnormal mitochondria characterized by dense staining and irregular shape. Bar: 500nm

**Fig 8:**
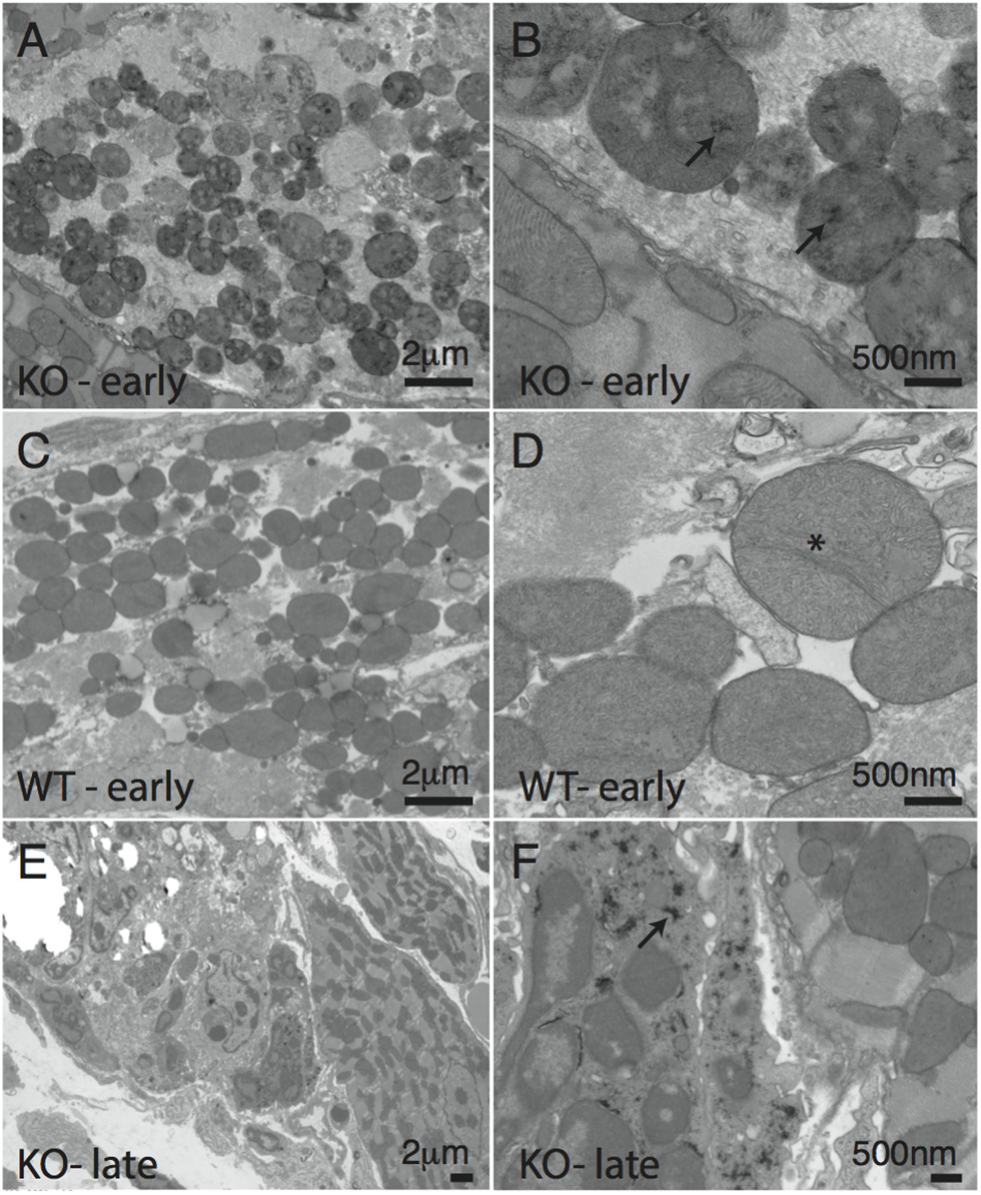
Genotype-dependent differences in mitochondrial morphology following infection. *Abcc6* KO (A,B,E,F) and WT (C,D) mice were infected with 50pfu/g CVB3 or mock infected with PBS. Mitochondria were characterized using transmission electron microscopy. At the early stage of necrosis, disruption of mitochondrial cristae was evident in both the *Abcc6* KO and WT mice (A-D). Fission and fusion abnormalities were also evident in both strains (D, asterisk). Electron dense deposits consistent with hydroxyapatite were only observed in *Abcc6* KO mice (A, B, arrow). At later stages of necrosis, the electron dense deposits became visible in extra-mitochondrial areas only in *Abcc6* KO mice (E, F, arrow).

Given the focal nature of the calcified lesions (Fig 2), we hypothesized that the swelling assay may not be sensitive enough to detect localized differences in mitochondrial function. Moreover, transient calcium fluxes from other organelles are known to impact the sensitivity of the mPTP and their effects cannot be measured in an *ex vivo* swelling assay. We therefore opted for an *in vivo* approach that would allow us to delineate affected and unaffected regions of an infected heart. Using transmission electron microscopy (TEM), we could define affected regions and identify genotype-dependent differences in mitochondrial morphology. Hearts from uninfected and infected (day 6 post infection) *Abcc6* WT and KO mice were excised, sectioned, and stained for TEM. No significantly discernible differences were observed between uninfected mice from the two genotypes (not shown). In infected mice, mitochondria within necrotic cells were visualized and compared. Given the dynamic nature of necrosis, we first outlined several temporal stages to ensure appropriate comparisons were made between genotypes (outlined in S5 Fig). The early stage was characterized by mitochondrial and plasma membrane intactness, the mid-stage by loss of mitochondrial intactness, and the late stage by loss of plasma membrane integrity. Mitochondria within necrotic foci were characterized according to several criteria: cristae disruption, fusion/fission events, presence of electron dense deposits consistent with hydroxyapatite, and abnormal shape (representative images in Fig 7). Extensive disruption of mitochondrial cristae and several fusion/fission abnormalities were evident in both strains of mice at the early and mid stages of necrosis (Table 1). Electron dense deposits consistent with hydroxyapatite, however, were observed predominantly in the mitochondria of *Abcc6* KO mice at the same stages (Fig 8A and 8B, Table 1). In the late stage, mitochondria were largely absent (S5 Fig). Those that remained were abnormally shaped and did not differ with genotype. The presence of electron dense deposits outside of mitochondria also became evident selectively in *Abcc6* KO mice in the late stage of necrosis (Fig 8E and 8F, Table 1). Altogether, these results highlight genotype-dependent morphological differences in mitochondria following infection. Moreover, they suggest that Abcc6 deficiency somehow renders mitochondria more susceptible to hydroxyapatite deposition, which is indicative of increased calcium concentration in the mitochondrial matrix.

**Table 1:** Genotype-dependent differences in mitochondrial morphology.

Stage of Necrosis		KO	WT	P-value^§^
Early*				
	Total Number of Foci	20	18	
	Disrupted Cristae	20	18	1.000
	Fusion / Fission	2	3	0.6525
	Hydroxyapatite-like deposits	18	0	**<0.0001**
Mid^†^				
	Total Number of Foci	55	65	
	Foci with Intact Mitochondria	54	64	
	Disrupted Cristae	54	64	1.000
	Fusion / Fission	4	1	0.1773
	Hydroxyapatite-like deposits	45	10	**<0.0001**
Late^‡^				
	Total Number of Foci	38	35	
	Foci with Intact Mitochondria	22	21	
	Abnormal Shape	20	17	0.8163
	Hydroxyapatite-like deposits	26	0	**<0.0001**

*Plasma membrane intact, majority of mitochondria intact (Fig 7A)

^†^Plasma membrane intact, diminished mitochondrial presence (Fig 7B)

^‡^Disruption of plasma membrane, lack of or very few mitochondria (Fig 7C)

^§^Chi-square test.

### Pharmacological inhibition of mPTP opening ameliorates CVB3-associated pathology

**Fig 9:**
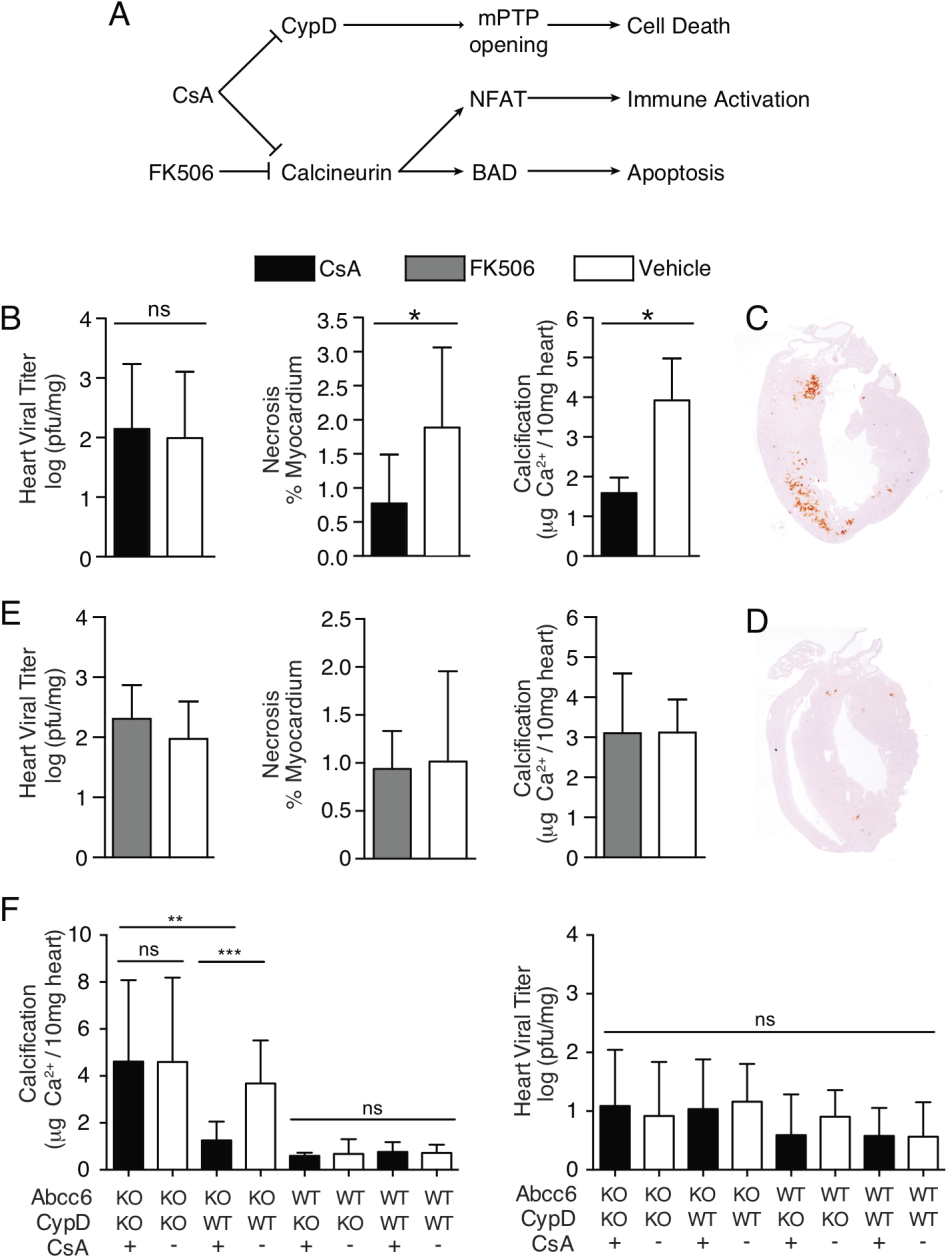
Inhibition of CypD in *Abcc6*-deficient mice prevents cardiac necrosis and calcification. (A) Schematic representing the molecular targets of CsA and FK506 and their downstream cellular effects. (B) Daily CsA (10mg/kg/day) treatment of *Abcc6* KO mice infected with 50pfu/g CVB3 attenuates CVB3-induced calcification and necrosis despite no effect on viral replication. (C, D) Representative images of vehicle treated (C) and CsA treated (D) hearts stained with alizarin, a calcium specific stain (red areas). Daily FK506 (1mg/kg/day) treatment of *Abcc6* KO mice infected with 50pfu/g CVB3 has no effect on cardiac viral titer, necrosis, or calcification (E). CsA treatment of *Abcc6* / *CypD* double knockout mice infected with 50pfu/g CVB3 has no effect on calcification or viral titer (F). The statistical test used for panels B and C was a student’s t test. The statistical test used for panel D was a two way ANOVA followed by Bonferonni post tests *: P<0.05, **: P<0.01, ***P<0.001.

Increased calcium concentrations in the mitochondrial matrix can initiate cellular necrosis by mPTP opening. Given that we observed increased necrosis in Abcc6 deficient mice despite equal viral replication, we hypothesized that pharmacologic inhibition of calcium-dependent mPTP opening could reduce cardiac necrosis and calcification in *Abcc6* KO mice. KO mice were treated daily with CsA (10mg/kg/day), an inhibitor of mPTP opening, or vehicle. Viral titer, necrosis, and calcification were evaluated at day 8 post infection. As observed previously[17], CsA treatment did not impact viral replication. It did, however, significantly decrease cardiac necrosis and calcification to less than half the values observed in vehicle-treated mice (Fig 9B, 9C and 9D).

CsA inhibits mPTP opening through its effects on CypD, a pore regulator. However, it also potently inhibits calcineurin, a signaling molecule that activates T cells upstream of nuclear factor of activated t cells (NFAT) and promotes apoptosis by dephosphorylating Bcl-2-associated death promoter (BAD) (outlined in Fig 9A). In order to exclude the effect of these interactions, we treated mice with FK506, a compound that inhibits calcineurin but not CypD. As FK506 is ten times as potent as CsA [34], mice were dosed at 1mg/kg/day and no significant differences in viral titer, necrosis, or calcification were observed (Fig 9E). Increasing the dose of FK506 to 10mg/kg/day had no impact on the experimental outcome (S6 Fig).

It is possible that CsA also interacts with as yet unidentified molecules and proteins. To exclude the potential role of these interactions in diminishing cardiac pathology, we treated *CypD* / *Abcc6* DKO and *Abcc6* SKO littermates with CsA. Whereas the effect of CsA on cardiac calcification remained evident in SKO mice, no effect was observed in DKO mice (Fig 9F), demonstrating that CypD inhibition is required for cardioprotection. Interestingly, though, untreated and vehicle-treated DKO mice were not protected from cardiac calcification when compared to SKO mice, suggesting that CypD may play several non-redundant roles in the heart (Fig 9F, S7 Fig). Nevertheless, these results show that CsA treatment potently diminishes Abcc6-dependent cardiac necrosis and calcification following CVB3 infection and that this occurs through its inhibitory effect on CypD.

## Discussion

We report that Abcc6 deficiency renders mice more susceptible to cardiac necrosis and calcification following CVB3 infection. Moreover, we show that inhibition of CypD, an mPTP regulator, by daily CsA treatment results in a significant reduction of both cardiac necrosis and calcification. The role of the mPTP in CVD and the role of the Abcc6 transporter in cardiac calcification have been explored in some depth [31, 35]. However, our study is the first to show a link between Abcc6-dependent cardiac calcification and the mPTP. Moreover, whereas others have explored the effectiveness of CsA treatment with conflicting results [36, 37], our study is the first to identify a genetic context wherein CsA treatment is reproducibly effective at countering CVB3-dependent cardiopathology. We therefore point to the value of targeting treatments according to both the genetic and environmental determinants of disease.

Cardiac calcification and inflammation were more severe in 129S1 mice than 129X1 mice upon infection with CVB3. Using a forward genetic approach, we showed that overlapping loci on chr. 7 controlled both traits. Interestingly, genetic complementation demonstrated that *Abcc6* was the gene underlying the calcification phenotype but not the inflammation phenotype. This may be due to the effect of another gene within the locus that independently controls inflammation. Indeed, White *et al* demonstrated that the 129X1 background harbors a genetic defect that impedes inflammatory cell recruitment [38]. They did not, however, identify a causative gene or genetic locus. Alternatively, lack of complementation of the inflammation phenotype may be due to the genetic background of the complementation animals (129xB6 F1). Several B6-specific myocarditis susceptibility loci have been identified [25], which may have affected the penetrance of *Abcc6* in our complementation crosses.

*Abcc6* encodes an ABC transporter expressed primarily in the liver and kidney [39] Human loss of function mutations cause pseudoxanthoma elasticum (PXE), a connective tissue disorder characterized by spontaneous and progressive mineralization of cardiovascular, ocular, and dermal tissues [39, 40]. Interestingly, the tissue localization and severity of symptoms differ between individuals, suggesting the involvement of other genetic and environmental determinants [41]. CVB3 infection, and perhaps viral infection generally, therefore represent environmental risk factors for PXE patients and may explain some of the inter-individual phenotypic variation.

Until very recently, the substrate of Abcc6 was unknown [21]. However, several independent studies had convincingly shown that the cardioprotective effect of Abcc6 was mediated by a circulating factor [42, 43]. Given that the mechanism underlying cardioprotection was unclear, we assessed the impact of Abcc6 deficiency on two important parameters of CVB3 infection: viral replication and cellular necrosis. Interestingly, cellular necrosis was increased in Abcc6 deficient compared to wild-type counterparts despite equal viral replication. Our results mirror those observed in a mouse model of ischemia reperfusion injury where the authors reported *Abcc6*-dependent differences in infarct size despite equal at-risk regions [44]. These observations suggest that Abcc6 somehow modulates the host’s response to exogenous insult. To identify potentially relevant host-response pathways, we evaluated transcriptomic signatures in the hearts of Abcc6 deficient and sufficient mice. We found genotype-specific differences in the expression of genes associated with mitochondria, suggesting that these organelles may play a role in mediating cardiopathology.

Mitochondria are important signaling hubs in the regulation of apoptosis and necrosis and calcium signaling is an important mediator of both of these. Large influxes of calcium into the mitochondrial matrix can initiate both apoptosis and necrosis via release of caspase cofactors and loss of membrane potential respectively [10, 28, 45]. We demonstrated an accumulation of hydroxyappatite in the mitochondria of *Abcc6* KO mice following infection. We also observed increased cell death in these mice. Together, these suggest that Abcc6 deficiency causes a defect in mitochondrial calcium signaling leading to increased cell death.

Very recently, it was shown by Jansen *et al* that ATP is a physiological substrate of Abcc6. ATP is transported out of hepatocytes, where it is rapidly metabolized to AMP and pyrophosphate [21]. Pyrophosphate enters the circulation where it inhibits ectopic deposition of calcium phosphate crystals [46]. Given that pyrophosphate is a charged molecule, it cannot cross the cellular membrane. As such, the mechanism whereby extracellular pyrophosphate might affect intracellular signaling remains unclear and merits further study. It is possible that another physiological substrate exists. Belinsky *et al* showed that Abcc6 can transport glutathione conjugates *in vitro* [47]. This may be relevant as glutathione is an antioxidant and large bursts of ROS have been observed during the course of CVB3 infection [9].

Increases in mitochondrial calcium are sensed by CypD, which then interacts with a component(s) of the mPTP to promote opening[28]. *CypD* KO mice have therefore been shown to be resistant to cardiopathology associated with calcium-induced mPTP opening[48]. Given that CsA inhibits Abcc6-dependent cardiac necrosis and calcification presumably through its interaction with CypD, one might expect DKO mice to be resistant to cardiac necrosis and calcification. However, no differences were observed between SKO mice and DKO mice. Several metabolic alterations have been observed in *CypD* KO mice that may explain this disconnect; changes in branched chain amino acid metabolism, pyruvate metabolism, the Krebs cycle, acylcarnitine metabolism, and the mitochondrial acetylome have been observed [49]. These changes may somehow promote cardiopathology. Indeed, Elrod *et al* have shown that *CypD* KO mice are more susceptible to cardiac pathology following exercise-induced stress as a result of metabolic alterations[50]. The efficacy of pharmacological inhibition over genetic ablation may be due to an incomplete inhibition by CsA as CsA may not inhibit all the functions of CypD. Alternatively, it may be the result of treatment longevity; functional loss of CypD is lifelong in knockout mice but only transient in CsA treated mice. Regardless, our data definitively indicate that CsA treatment inhibits cardiac necrosis and calcification following infection and that this protective effect occurs through its inhibition of CypD.

Treatment with CsA may be ill-advised due to its immunosuppressive properties. However, specific inhibitors of CypD may prove therapeutically beneficial. Debio 025 is a CsA derivative that lack immunosuppressive properties but still block mPTP opening by inhibiting CypD [51]. Currently, Debio 025 is in stage 3 clinical trials for the treatment of hepatitis C, making it an ideal candidate for drug repurposing [52]. Moreover, the clinical significance may be broader than just the treatment of CVB3-induced pathology. Soft tissue calcification is observed in several human diseases. Deleterious mutations in human *ABCC6*, *ENPP1*, *NT5E* genes cause PXE, generalized arterial calcification of infancy (GACI), and arterial calcification respectively [39, 53, 54]. Interestingly, Martin *et al* have recently described mitochondrial defects in their model of PXE [55]. Given the overlapping genetic etiologies and phenotypic similarities, inhibition of CypD may be broadly therapeutic for several disorders associated with soft tissue calcification.

## Supporting Information

S1 ARRIVE Checklist NC3Rs ARRIVE Guildelines Checklist. (PDF)

S1 Fig Genetic diversity in 129S1 and 129X1 mice. (DOCX)

S2 Fig Confirming viral titer and cellular necrosis observations in *Abcc6* KO and WT/Het mice. (DOCX)

S3 Fig Common transcriptional signatures of coxsackievirus infection. (DOCX)

S4 Fig *Ex vivo* mitochondrial swelling assays to evaluate the kinetics of mPTP opening. (DOCX)

S5 Fig Outlining stages of necrosis for comparative TEM. (DOCX)

S6 Fig FK506 (10mg/kg/day) treatment has no effect on cardiac calcification or viral titer. (DOCX)

S7 Fig Cardiac calcification and heart viral titer in untreated *Abcc6* / *CypD* double knockout mice. (DOCX)

S1 Table Number of mice per experiment (DOCX)

S2 Table SNP IDs and genomic locations used in to genotype [129X1 x 129S1] F2 mice. (DOCX)
