# Supplementary material for: Cyclosporine A Treatment Inhibits Abcc6-Dependent Cardiac Necrosis and Calcification following Coxsackievirus B3 Infection in Mice
Source: PLoS One. 2015 Sep 16;10(9):e0138222. doi: 10.1371/journal.pone.0138222 (PMC4574283; doi:10.1371/journal.pone.0138222)
Supplement: S1 Fig — (DOCX) [file pone.0138222.s002.docx]

S1 Fig: Genetic diversity in 129S1 and 129X1 mice. 129S1 and 129X1 mice were genotyped at 600 thousand genetic loci using JAX’s 600K Diversity Chip. The horizontal lines represent SNPs that are polymorphic between the two strains.
