# Supplementary material for: Cyclosporine A Treatment Inhibits Abcc6-Dependent Cardiac Necrosis and Calcification following Coxsackievirus B3 Infection in Mice
Source: PLoS One. 2015 Sep 16;10(9):e0138222. doi: 10.1371/journal.pone.0138222 (PMC4574283; doi:10.1371/journal.pone.0138222)
Supplement: S2 Fig — (DOCX) [file pone.0138222.s003.docx]

S2 Fig: Confirming viral titer and cellular necrosis observations in *Abcc6* KO and WT/Het mice. Increased necrosis was also observed in infected hearts from *Abcc6* KO mice compared to Het and WT counterparts (A). This occurred despite equal viral replication (B). The statistical test used was a student’s t test. *: P<0.05, ns: not significant.
