# Supplementary material for: Cyclosporine A Treatment Inhibits Abcc6-Dependent Cardiac Necrosis and Calcification following Coxsackievirus B3 Infection in Mice
Source: PLoS One. 2015 Sep 16;10(9):e0138222. doi: 10.1371/journal.pone.0138222 (PMC4574283; doi:10.1371/journal.pone.0138222)

S3 Fig: Common transcriptional signatures of coxsackievirus infection. (A) Principal component analysis of the microarray data shows that mice in different experimental groups cluster together. Genotype and infection account for 10.83% and 59.14% of expression variances respectively. (B) Gene expression changes that occur in both 129S1 and 129X1 mice with infection were determined and are illustrated by heatmap. (C) Functional clustering of these genes using DAVID identified significant enrichment of several clusters associated with potent activation of immune, defense, and inflammatory responses. Bars represent the enrichment scores and points represent the –log(Bonferonni pvalue) as determined by DAVID.


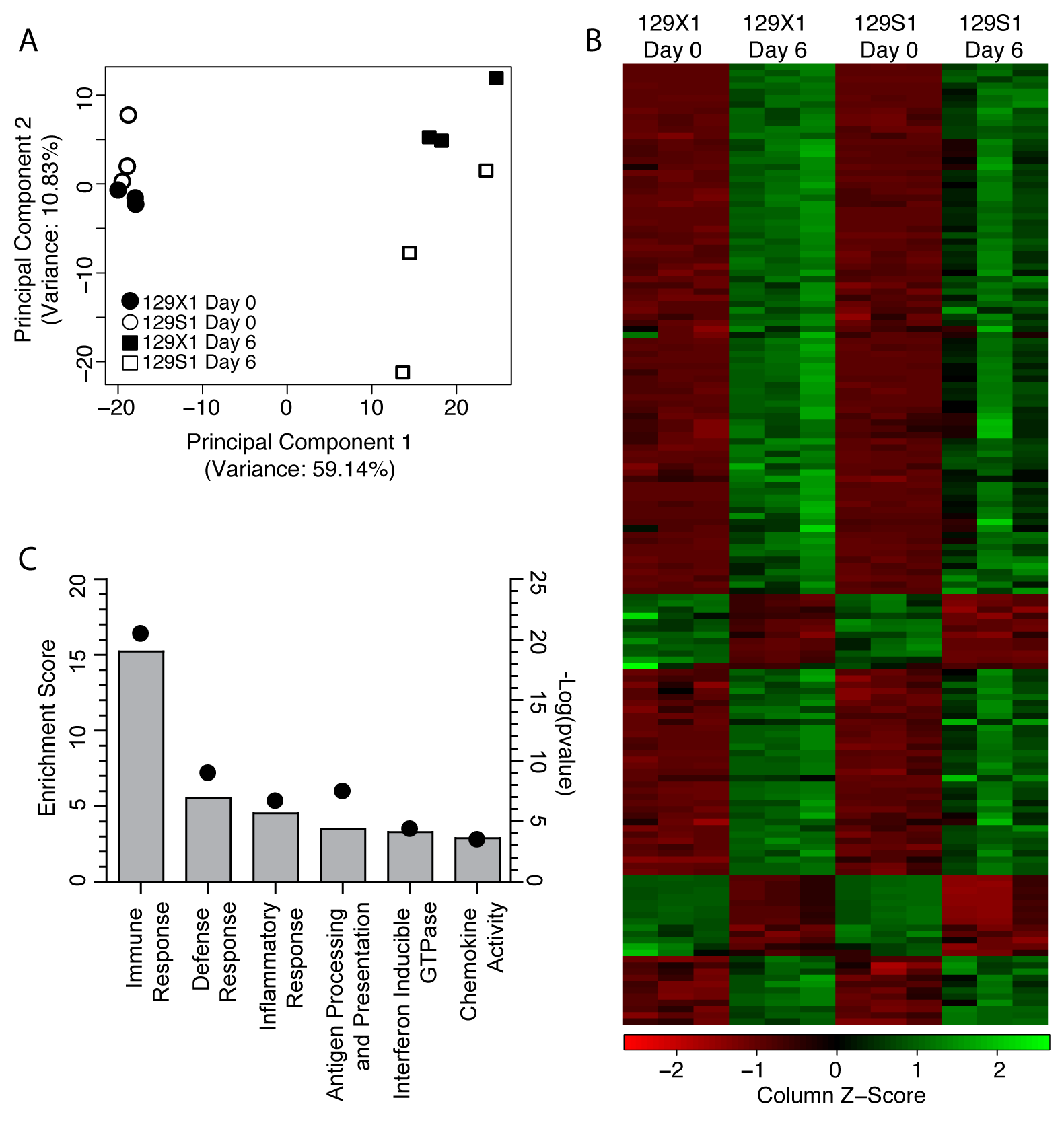

Supplement: S3 Fig — (DOCX) [file pone.0138222.s004.docx]
