# Supplementary material for: Cyclosporine A Treatment Inhibits Abcc6-Dependent Cardiac Necrosis and Calcification following Coxsackievirus B3 Infection in Mice
Source: PLoS One. 2015 Sep 16;10(9):e0138222. doi: 10.1371/journal.pone.0138222 (PMC4574283; doi:10.1371/journal.pone.0138222)
Supplement: S4 Fig — (DOCX) [file pone.0138222.s005.docx]

S4 Fig: *Ex vivo* mitochondrial swelling assays to evaluate the kinetics of mPTP opening. Mitochondria from uninfected and infected *Abcc6* KO and WT mice were isolated and incubated with either 0μM (A) or 400μM (B) CaCl_2_. Changes in optical density indicative of mPTP opening were measured. No genotype-dependent differences in mPTP opening were observed.
