# Supplementary material for: Cyclosporine A Treatment Inhibits Abcc6-Dependent Cardiac Necrosis and Calcification following Coxsackievirus B3 Infection in Mice
Source: PLoS One. 2015 Sep 16;10(9):e0138222. doi: 10.1371/journal.pone.0138222 (PMC4574283; doi:10.1371/journal.pone.0138222)

S5 Fig: Outlining stages of necrosis for comparative TEM studies. Areas of necrosis were determined using brightfield microscopy of toluidene stained thick sections (white outline in A). Early (B, black outline), mid (C, black outline), and late (D, black outline) stages of necrosis were outlined based on transmission electron microscopy imaging. The early stage was characterized by complete loss of sarcolemmal structure but maintenance of mitochondrial integrity. The mid stage was characterized by the maintenance of plasma membrane integrity but by a large loss of mitochondrial integrity. The late stage was characterized by the loss of plasma membrane integrity.


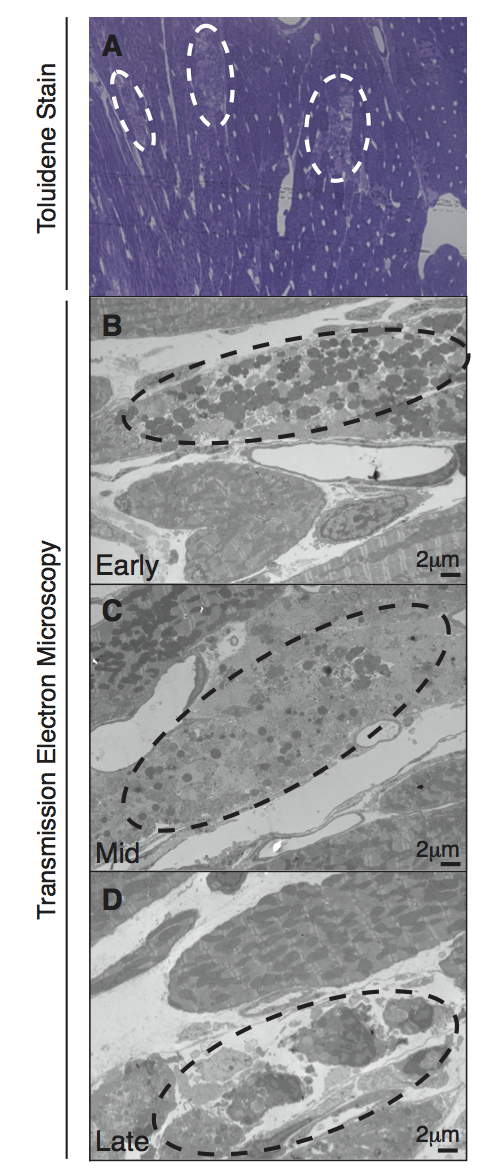

Supplement: S5 Fig — (DOCX) [file pone.0138222.s006.docx]
