# Supplementary material for: Cyclosporine A Treatment Inhibits Abcc6-Dependent Cardiac Necrosis and Calcification following Coxsackievirus B3 Infection in Mice
Source: PLoS One. 2015 Sep 16;10(9):e0138222. doi: 10.1371/journal.pone.0138222 (PMC4574283; doi:10.1371/journal.pone.0138222)

S6 Fig: *Abcc6* KO mice were treated daily with 10mg/kg/day FK506 or vehicle and cardiac calcification and viral titer were evaluated 8 days following infection with 50pfu/g CVB3. Treatment had no effect on either phenotype. The statistical test used was a student’s t test. ns: not significant.


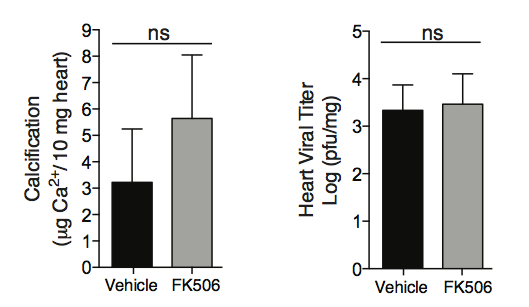

Supplement: S6 Fig — (DOCX) [file pone.0138222.s007.docx]
