# Supplementary material for: Cyclosporine A Treatment Inhibits Abcc6-Dependent Cardiac Necrosis and Calcification following Coxsackievirus B3 Infection in Mice
Source: PLoS One. 2015 Sep 16;10(9):e0138222. doi: 10.1371/journal.pone.0138222 (PMC4574283; doi:10.1371/journal.pone.0138222)

S7 Fig: Cardiac calcification and viral titer in Abcc6^+/+^ / CypD^+/+^, Abcc6^+/+^ / CypD^-/-^, Abcc6^-/-^ / CypD^+/+^, Abcc6^-/-^ / CypD^-/-^ mice following infection with 50pfu/g .


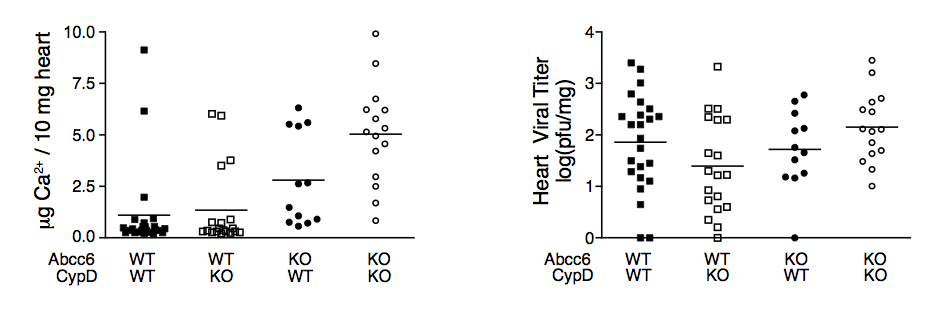

Supplement: S7 Fig — (DOCX) [file pone.0138222.s008.docx]
