# Supplementary figures and images for: Cyclosporine A Treatment Inhibits Abcc6-Dependent Cardiac Necrosis and Calcification following Coxsackievirus B3 Infection in Mice
Source: PLoS One. 2015 Sep 16;10(9):e0138222. doi: 10.1371/journal.pone.0138222 (PMC4574283; doi:10.1371/journal.pone.0138222)

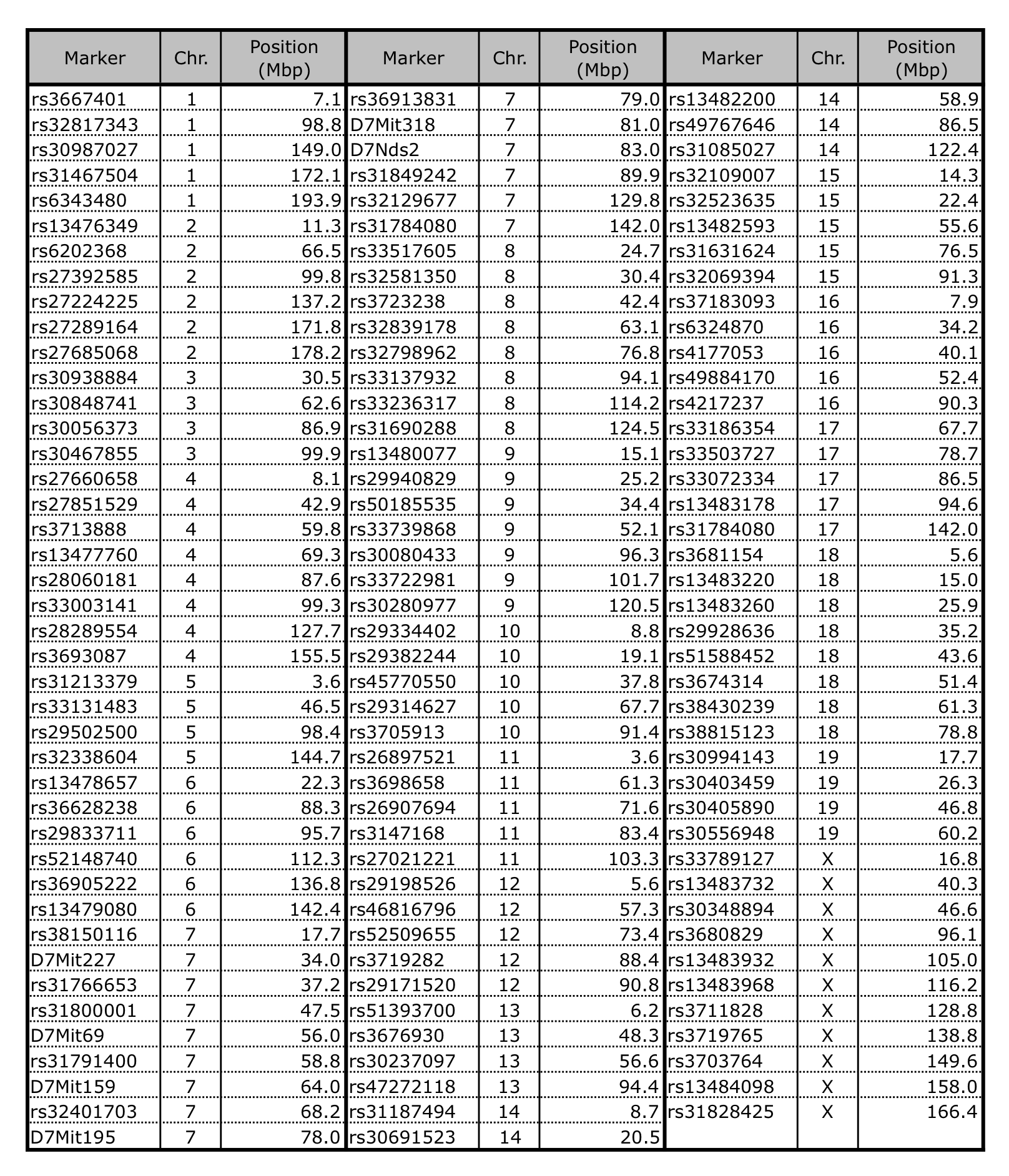
S2 Fig:

Supplement: S2 Table — (DOCX) [file pone.0138222.s010.docx]
